# Chylous Ascites in a Patient with HIV/AIDS: A Late Complication of *Mycobacterium avium* Complex-Immune Reconstitution Inflammatory Syndrome

**DOI:** 10.1155/2014/268527

**Published:** 2014-11-18

**Authors:** Imam H. Shaik, Fernando Gonzalez-Ibarra, Rumana Khan, Saira Shah, Amer K. Syed, David Lintz

**Affiliations:** ^1^Department of Internal Medicine, Jersey City Medical Center, 355 Grand Street, Jersey City, NJ 07302, USA; ^2^Laureate National Institute of Medicine, Program Director Internal Medicine, Jersey City Medical Center, Jersey City, NJ 07302, USA; ^3^Department of Infectious Diseases, Jersey City Medical Center, 355 Grand Street, Jersey City, NJ 07302, USA

## Abstract

Chylous ascites is very rare in HIV/AIDS and its association with *Mycobacterium avium* complex-immune reconstitution inflammatory syndrome (MAC-IRIS) has been rarely reported. Here, we report a case of a young African-American male who developed chylous ascites as a late sequela to immune reconstitution inflammatory syndrome while on treatment for MAC. Antiretroviral drug-naive patients who start HAART in close proximity to the diagnosis of an opportunistic infection and have a rapid decline in HIV RNA level should be monitored for development of IRIS. Although the long term prognosis is poor, early diagnosis and treatment help to improve quality of life.

## 1. Introduction

Chylous ascites is defined as the accumulation of triglyceride rich fluid in the peritoneal cavity [1]. It is an exceedingly rare condition usually caused by obstruction or rupture of the peritoneal or retroperitoneal lymphatic channels. Among HIV-positive patients, chylous ascites has been rarely reported in association with* Mycobacterium tuberculosis* (MTB),* Mycobacterium avium* complex (MAC), lymphoma, Kaposi sarcoma, and/or as a complication of immune reconstitution inflammatory syndrome (IRIS). Herein we report a case of young HIV-positive male with disseminated MAC infection, who developed chylous ascites despite of his improving CD4 count and downtrending of HIV RNA log copies, suggestive of immune reconstitution inflammatory syndrome (IRIS).

## 2. Case Description

A 44-year-old HIV infected African-American male presented with three-month history of progressive abdominal distension, chronic backache, and exertional shortness of breath. Otherwise the patient denied of any fever, cough, sputum production, weight loss, night sweats, paroxysmal nocturnal dyspnea, or jaundice. His past medical history is significant for three-year history of HIV/AIDS and six-month history of disseminated MAC of duodenum and bone marrow. He has been compliant with his antiretroviral treatment (ART) and MAC regimen. His current medications include ritonavir, darunavir, emtricitabine/tenofovir, rifabutin, enfuvirtide, azithromycin, ethambutol, and atovaquone.

**Figure 1:**
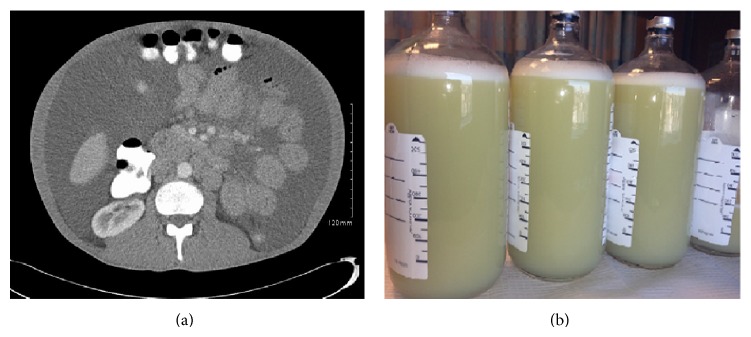
(a) Contrast CT of abdomen and pelvis showing extensive ascites with mesenteric and retroperitoneal lymphadenopathy. (b) Gross specimen of paracentesis showing thick pale yellowish chylous fluid.

Physical examination is consistent with mild respiratory distress on supine position. Abdominal examination revealed large ascites without any signs of portal hypertension. He is afebrile and hemodynamically stable. Abdominal imaging showed extensive ascites, hepatosplenomegaly, and mesenteric and retroperitoneal adenopathy (Figure 1(a)). Diagnostic and therapeutic paracentesis was done with a removal of five liters of thick pale yellow grossly turbid fluid (Figure 1(b)) which is a transudate according to Light's criteria. Fluid analysis: glucose: 98 mg/dL, neutrophils: 18%, lymphocytes: 31%, monocytes: 51%, amylase: 37 U/L, fluid LDH: 196 IU/L, fluid total protein: 6.1 g/dL, albumin: 2.3 g/dL, triglycerides: 239 mg/dL, serum LDH: 362 IU/L, serum total protein: 8.7 gm/dL, serum albumin: 3.5 g/dL, and serum triglycerides: 118 mg/dL. Cytology was negative for malignant cells; Gram stain and cultures were negative for bacteria, fungus, and acid-fast bacilli after six weeks. The diagnosis of chylous ascites was made based on visual appearance of fluid and triglyceride level of more than 110 mg/dL.

The absolute CD4 count and HIV viral load at the time of MAC diagnosis were 103 cells/mcL and 4.99 log copies/mL, respectively. The progressive improvement in absolute CD4 counts from 90 cells/mcL, 103 cells/mcL, 130 cells/mcL, and 222 cells/mcL and downtrending HIV-1 RNA PCR log copies from 4.99 to 2.42 to 1.30 in six-month intervals despite his worsening clinical condition is consistent with immune reconstitution inflammatory syndrome (IRIS) in our patient.

## 3. Discussion

Chylous ascites is an abnormal accumulation of triglyceride rich lymphatic fluid in the peritoneal cavity. This occurs usually as a result of lymphatic leakage or obstruction of lymphatic drainage in the peritoneal cavity. It is very rare in general population with an approximate incidence of 1 in 12,000 hospital admissions before HIV era. The most common causes listed were malignancy, cirrhosis, surgery and tuberculosis [1]. Few cases of chylous ascites in HIV/AIDS were reported in literature; most of them were related with* Mycobacterium tuberculosis* (MTB),* Mycobacterium avium* complex (MAC), Kaposi sarcoma (KS), and lymphoma. A case series published by Phillips et al. from a single institution reported an incidence rate of 1 in 2248 HIV-positive admissions; all of them were diagnosed with intra-abdominal MAC prior to developing chylous ascites similar to our case [2].

The diagnosis of chylous ascites is made by analyzing the fluid obtained through paracentesis. Among others, usual findings that point towards the identification of this condition are milky white ascetic fluid with triglyceride levels greater than 110 mL/dL and high leukocyte count of mononuclear cell predominance [1]. Chylous ascites, in association with HIV and MAC is rarely reported and its pathology is unique as is our case, when compared to other historical causes which are mostly traumatic during surgery.

Most often, this pathological process develops secondary to a complication of abdominal surgeries, in which the major retroperitoneal lymphatic flow is interrupted due to trauma to the lymphatic system [1]. The cisterna chyli is situated between the posterior aspect of the abdominal aorta and vertebral bodies of L1-L2. Obstruction to this lymphatic trunk at the level of the abdomen in our case was most probably secondary to granulomatous infiltration, resulting into chyloperitoneum, and presents with symptoms of progressive abdominal distention, dyspnea, abdominal pain, steatorrhea, edema, and paralytic ileus. Other causes associated with the development of chyloperitoneum include malignancy, cirrhosis, and the development of ascites secondary to nephritic syndrome [1]. In the setting of* Mycobacterium tuberculosis *and* Mycobacterium avium *complex (MAC) infection, histologically well-formed granulomas on lymphatics suggest that the pathogenesis of chylous ascites is presumed to be related to granulomatous lymphadenitis causing lymphatic obstruction at the base of the mesentery or the cisterna chyli, analogous to cases associated with neoplastic infiltration and fibrosis [2, 3].

The immune reconstitution inflammatory syndrome (IRIS) complicates the preexisting infections in HIV infected patients, in whom antiretroviral therapy (ART) was initiated early in the course, especially with low CD4 count at the beginning of treatment. This pathological process results from restoring immunity to specific infectious or noninfectious antigens [4]. The abrupt restoration of the immune system induces an excessive inflammatory reaction that promotes tissue damage. Upon this rapid reactivation of the immunity, CD4+ Th1 cells release IFN-*γ*, activate macrophages, and stimulate natural killer cell induced cytotoxicity, promoting a proinflammatory response. Additionally, in the case of concomitant mycobacterium infection, the initiation of antituberculous therapy inhibits the immunosuppressive effect of CD4+ Th2, further exacerbating the inflammatory response. It is estimated that IRIS occurs in at least 10% of patients with HIV who started on ART concomitantly with tuberculosis treatment and it is seen commonly in patients with low numbers of CD4 cells. The systemic and inflammatory reaction usually presents 3-4 weeks after the introduction of ART and is associated with reductions in the viral load and increase in the CD4+ T lymphocyte count. The delayed onset of symptoms can occur as in our case. It initially presents with worsening symptoms of the particular system involved and increased lymphadenopathy, and it is self-limited in most of the cases, especially if the underlying infection is effectively treated, but fatal complications present in some cases.

The management of this condition appears to be associated with a generally favorable prognosis with the use of specific HIV and MAC therapy [5]. Typically, these patients need repeated therapeutic paracentesis to relieve intra-abdominal pressure, low-fat diet, and medium-chain triglyceride supplementation and reserving total parenteral nutrition for patients that cannot tolerate oral intake to reduce the flow of chyle into the lymphatic system. The use of somatostatin analogues (octreotide) has been proven effective in reducing lymphorrhagia. Surgery is reserved for large lymph vessel leakage confirmed by lymphangiography, which helps to localize the site of leaking as well. Finally, peritoneovenous shunts are suggested for refractory cases.

## 4. Conclusion

Chylous ascites in patients with HIV complicated with disseminated MAC infection is a rare condition that needs to be assessed and treated in the early presentation because of its high mortality and morbidity. The prognosis depends on the management of primary disease rather than the condition itself. Finally, the danger of developing infection and nutritional disturbances is a feared complication; early intervention and nutritional support are necessary. In several reports, a brief course of systemic prednisone (e.g., 1 mg/kg/day for 1-2 weeks followed by a slow taper) appeared to be of benefit for severe cases of mycobacterial IRIS.

## Abbreviations

HIV: Human immunodeficiency virus
MAC: * Mycobacterium avium* complex, also known as* Mycobacterium avium*-intracellulare
IRIS: Immune reconstitution inflammatory syndrome.
